# Incidence and Management of Adverse Events Associated with Tebentafusp Treatment in Metastatic Uveal Melanoma: Pooled Safety Analysis of 410 Patients

**DOI:** 10.1158/1078-0432.CCR-25-1513

**Published:** 2025-09-12

**Authors:** Takami Sato, Marcus O. Butler, Sophie Piperno-Neumann, Jessica C. Hassel, Paul Nathan, Alexander N. Shoushtari, Piotr Rutkowski, Richard D. Carvajal, Jean-Francois Baurain, Max Schlaak, Josep M. Piulats, Ryan J. Sullivan, Sebastian Ochsenreither, Reinhard Dummer, John M. Kirkwood, Alexandra Ikeguchi, Anthony M. Joshua, Mark R. Middleton, Ramakrishna Edukulla, Constance Pfeiffer, Joseph J. Sacco

**Affiliations:** 1 Sidney Kimmel Cancer Center, Jefferson University, Philadelphia, Pennsylvania.; 2 Princess Margaret Cancer Centre, University Health Network, Toronto, Canada.; 3 Departments of Medicine and Immunology, University of Toronto, Toronto, Canada.; 4 Institut Curie, PSL Research University, Paris, France.; 5 Heidelberg University, Medical Faculty Heidelberg, Department of Dermatology and National Center for Tumor Diseases (NCT), NCT Heidelberg, a partnership between DKFZ and University Hospital Heidelberg, Heidelberg, Germany.; 6 Mount Vernon Cancer Centre, Northwood, Middlesex, United Kingdom.; 7 Memorial Sloan Kettering Cancer Center, New York, New York.; 8 Maria Sklodowska-Curie National Research Institute of Oncology, Warsaw, Poland.; 9 Northwell Health Cancer Institute, New York, New York.; 10 Cliniques universitaires Saint-Luc, UCLouvain, Brussels, Belgium.; 11 Charité - Universitätsmedizin Berlin, corporate member of Freie Universität Berlin, Humboldt-Universität zu Berlin, Department of Dermatology, Venereology and Allergology, Berlin, Germany.; 12 Institut Català d’Oncologia, Barcelona, Spain.; 13 Massachusetts General Hospital Cancer Center, Boston, Massachusetts.; 14 Charité – Universitätsmedizin Berlin, corporate member of Freie Universität Berlin, Humboldt-Universität zu Berlin, and Berlin Institute of Health; Department of Hematology, Oncology and Tumor immunology and Comprehensive Cancer Center, Berlin, Germany.; 15 University of Zürich Hospital, Zürich, Switzerland.; 16 Kantonsspital Aarau, Aarau, Switzerland.; 17 University of Pittsburgh Medical Center UPMC Cancer Center, Pittsburgh, Pennsylvania.; 18 Department of Melanoma Medical Oncology, The University of Texas MD Anderson Cancer Center, Houston, Texas.; 19 Kinghorn Cancer Centre, Saint Vincent’s Hospital, Darlinghurst, Australia.; 20 Medical Sciences Division, Department of Oncology, University of Oxford, Oxford, United Kingdom.; 21 Immunocore, Gaithersburg, Maryland.; 22 Immunocore, Conshohocken, Pennsylvania.; 23 The Clatterbridge Cancer Centre NHS Foundation Trust, Bebington, Wirral, United Kingdom.; 24 University of Liverpool, Liverpool, United Kingdom.

## Abstract

**Purpose:**

We conducted an integrated safety analysis from three clinical studies of tebentafusp, a first-in-class ImmTAC bispecific T-cell engager, which can redirect T cells to target glycoprotein 100–positive cells, in metastatic uveal melanoma.

**Experimental Design:**

HLA-A*02:01–positive patients with unresectable or metastatic uveal melanoma enrolled in three clinical trials (IMCgp100-01, IMCgp100-102, and IMCgp100-202) who received ≥1 dose of tebentafusp were included. Safety data were pooled to evaluate the profile, onset, and management of treatment-related adverse events (TRAE). Adverse events of special interest included cytokine release syndrome (CRS), acute skin reactions (ASR), and liver function test elevations. Primary prophylaxis with medications was not permitted.

**Results:**

Among 410 tebentafusp-treated patients, the most common TRAE were pyrexia (77%), pruritus (71%), and chills (53%). Most patients experienced CRS (88%), almost always mild (grade 1, 19%) to moderate (grade 2, 67%) in severity, with only 2% experiencing grade 3 (*n* = 6) or 4 (*n* = 1) CRS. Additionally, 92% had at least one ASR, primarily pruritus and rash, with 21% having a grade 3 event. Onset of CRS and ASR was within 1 to 2 days of infusion and generally reversible with standard interventions. Elevated liver function tests were generally mild and resolved without intervention. Most TRAE occurred following the first few infusions and diminished in frequency and severity with repeated dosing; no cumulative TRAE were detected. Discontinuations due to TRAE were rare (2%); there were no treatment-related deaths.

**Conclusions:**

TRAE were consistent with tebentafusp’s mechanism of action, mostly occurred during dose escalation, and were predictable, reversible, and manageable with appropriate surveillance and intervention.

**Translational Relevance**

Tebentafusp is a first-in-class ImmTAC bispecific T-cell engager. As tebentafusp utilization increases in clinical practice and new TCR bispecifics enter clinical trials, a comprehensive understanding of the safety profile of this new class of immunotherapy is important for the timely detection and effective management of related adverse events (AE). We report a pooled safety analysis of data from three clinical trials of tebentafusp in patients with metastatic uveal melanoma to provide detailed information on the incidence, time to onset, and clinical management. The data reported herein show that tebentafusp has a predictable and manageable safety profile, with most treatment-related AE being consistent with T-cell activation (e.g., cytokine release syndrome) or redirection of T cells to the skin (e.g., rash), with no significant evidence of off-target or long-term toxicity. In addition to guiding clinical management, the data will help the investigation of future TCR bispecifics and provide insights into the mechanism of action and timing/evolution of AE.

## Introduction

Tebentafusp is the only systemic therapy to date to demonstrate an overall survival (OS) benefit in metastatic uveal melanoma (mUM) and is the first T-cell receptor (TCR) bispecific approved for clinical use (1–3). Tebentafusp also represents a new class of T-cell–engaging immunotherapies. Although other T-cell–engaging therapies have demonstrated efficacy in hematologic cancers (4), the immune-mobilizing monoclonal TCR against cancer (ImmTAC) platform is the first T-cell engager to demonstrate an OS benefit in a solid tumor.

ImmTAC molecules, including tebentafusp, can target any intra- or extracellular protein processed and presented as peptide fragments by HLA molecules on the cancer cell surface. This enables the targeting of intracellular proteins that have higher specificity for malignant cells and limits off-tumor toxicity to non-vital tissue (3, 5). Tebentafusp comprises a soluble affinity-enhanced TCR (pmol/L affinity) fused to an anti-CD3 T-cell–activating domain (nmol/L affinity). It redirects polyclonal T cells to target melanoma cells, and normal melanocytes, presenting a specific glycoprotein 100 (gp100) peptide (YLEPGPVTA) by HLA-A*02:01 on the cell surface (6, 7).

Uveal melanoma is the most common primary intraocular malignancy in adults (8). Despite localized ocular therapy to preserve vision and prevent distant metastasis, up to half of patients develop distant disease, with initial metastases primarily developing in the liver (9).

Tebentafusp was first studied in advanced melanoma (IMCgp100-01, including 19 patients with uveal melanoma) and subsequently in mUM (IMCgp100-102 and IMCgp100-202; refs. 1, 10–12). The most frequent treatment-related adverse events (TRAE) included cytokine-mediated (e.g., pyrexia and hypotension) and skin (e.g., rash and pruritus) toxicities. Both classes of TRAE were expected based on the mechanism of action of tebentafusp. Cytokine release syndrome (CRS), characterized by systemic release of cytokines, is a TRAE observed for all therapies that rapidly activate T cells, including T-cell engagers and autologous chimeric antigen receptor T-cell therapies (13, 14). Skin adverse events (AE) such as rash and pruritus are thought to be an on-target, off-tumor TRAE resulting from T-cell redirection to skin melanocytes that also express gp100, albeit at much lower levels than in melanoma (15). Nearly all patients with mUM have liver metastases and bystander inflammation and local cytokine release are potential mechanisms of liver AE; therefore, hepatic TRAE were also considered an AE of special interest (AESI).

As tebentafusp utilization increases in clinical practice and new TCR bispecifics enter clinical trials, it is imperative that health care providers are equipped with the knowledge to effectively manage AE associated with this emerging class of T-cell engagers. This analysis aimed to comprehensively evaluate the profile of TRAE and AESI using pooled data from all tebentafusp-treated patients with mUM included in the IMCgp100-01, IMCgp100-102, and IMCgp100-202 trials.

## Materials and Methods

### Study design

Data from 410 patients across three studies were pooled: IMCgp100-01 (*n* = 19; cutoff date August 11, 2017), IMCgp100-102 (*n* = 146; cutoff date March 12, 2020), and IMCgp100-202 (*n* = 245; cutoff date October 13, 2020); study details have been previously reported (1, 10–12). All studies adhered to the Declaration of Helsinki and the International Council for Harmonization Good Clinical Practice guidelines. Protocols were approved by the local institutional review board or independent ethics committee, and all patients provided written informed consent.

IMCgp100-01 was a first-in-human, open-label, dose-finding phase I/II study in stage IV or unresectable stage III melanoma (NCT01211262; ref. 12), including 19 patients with mUM who received daily or weekly tebentafusp (10–50 μg/day or 5–900 ng/kg/week). IMCgp100-102 was an open-label phase I/II mUM study (NCT02570308) with a phase I dose escalation (*n* = 19; 20 μg week 1, 30 μg week 2, and 54–73 µg from week 3; ref. 10) and a phase II expansion (*n* = 127; 20 μg week 1, 30 μg week 2, and 68 µg weekly thereafter; ref. 11). IMCgp100-202 was an open-label, phase III trial (NCT03070392) in previously untreated HLA-A*02:01–positive patients with mUM (*n* = 245, safety population). Patients received intravenous tebentafusp at a dose of 20 μg on day 1, 30 μg on day 8, and 68 μg weekly thereafter (1). Patients were monitored after infusion for 2 hours in IMCgp100-01 and for 16 hours after each of the first three weekly doses (and week 4 if prior hypotension occurred in the previous week) in IMCgp100-102/-202 to manage hypotension and other cytokine-related AE. Primary prophylaxis with corticosteroids, antihistamines, or acetaminophen was not permitted; secondary prophylaxis was permitted for certain AE (e.g., antihistamines after grade 3 rash; corticosteroids for recurrent hypotension unresponsive to antipyretics and intravenous fluids).

In IMCgp100-102 and IMCgp100-202, treatment continued until disease progression, unacceptable toxicity, investigator decision, or patient withdrawal of consent. Patients who experienced disease progression per RECIST version 1.1 could continue therapy beyond initial radiographic progression if they did not have symptomatic progression requiring alternative therapy and the investigator believed they were continuing to derive clinical benefit. Treatment could be continued for patients meeting these criteria. Therapy was stopped if, at least 4 weeks after initial progressive disease, one of the following occurred: an additional ≥20% increase in tumor burden from the new baseline (sum of diameters of target plus new measurable lesions) with an absolute increase of ≥5 mm, unequivocal progression of nontarget lesions, or the appearance of new nonmeasurable lesions.

### Patients

Eligibility for all patients in this analysis included histologic or cytologic confirmation of mUM, age ≥18 years, HLA-A*02:01 positivity, and Eastern Cooperative Oncology Group (ECOG) performance status 0 or 1. IMCgp100-01 and IMCgp100-102 permitted any number of prior therapies (10–12). IMCgp100-202 enrolled patients without prior systemic or liver-directed therapy (other than surgery) for metastatic disease (1).

### Endpoints and assessments

AE were coded using the Medical Dictionary for Regulatory Activities v23.1 or later and graded per NCI Common Terminology Criteria for Adverse Events (CTCAE), v4.03; original data were recoded where necessary. Treatment-emergent AE were defined as any AE from first dose up to 90 days after last dose or until the initiation of alternative cancer therapy after discontinuation. Causality between the study drug and/or concomitant therapy and the AE was assessed by the investigator. AESI included CRS, acute skin reactions (ASR), and elevation in liver function tests (LFT).

Medications were coded using World Health Organization Drug Dictionary (01MAR2019). In IMCgp100-102/-202, medications were linked to the AE as reported by the investigator; in IMCgp100-01, medication data were not directly linked and were excluded from that summary.

CRS in IMCgp100-102 and IMCgp100-202 was graded *post hoc* per the 2019 American Society for Transplantation and Cellular Therapy Consensus (ASTCT) Grading (16). Key data, such as oxygen usage, were not collected in IMCgp100-01; thus, patients from this study were excluded from CRS analyses.

ASR comprised the major subcategories of rash, erythema, cutaneous edema, and pruritus. Melanocyte-related AE (MRAE) is a composite term for a list of related hypo-/hyperpigmentation events. The standardized Medical Dictionary for Regulatory Activities query for “Drug-related hepatic disorders – comprehensive search” identified LFT events for broad- and narrow-scope terms; only narrow-scope terms were used in these analyses. Additional analyses using the LFT laboratory data were also conducted. Preferred terms for each composite are listed in Supplementary Table S1.

Hospitalization was defined as any unplanned hospitalization and, for the tebentafusp arm, also included any hospitalization during dose escalation that was prolonged for >2 days. Serious AE (SAE) were defined as occurrences that required inpatient hospitalization or prolongation of existing hospitalization, were fatal or life-threatening, resulted in persistent or significant disability, were a congenital anomaly/birth defect, or any event that posed a substantial risk to the patient or necessitated medical or surgical intervention.

Peripheral cytokines were assessed in IMCgp100-102 from peripheral blood samples taken before treatment and 8 hours after the first dose (*n* = 142). Serum IFN-γ, IL-10, and IL-6 were measured using a MILLIPLEX MAP Custom Magnetic Bead Panel (EDM Millipore; SPRCUS423).

### Statistical analyses

All patients in the safety populations who received ≥1 dose of tebentafusp were included. Data are shown as *n* (%), median (range), mean (SD), or mean (SEM). Incidents were also reported by weekly/monthly periods (e.g., days 1–7, 8–14, etc.), for which repeat events with new onsets were considered for each interval. For laboratory and vital sign evaluations, the minimum and/or maximum values within each week were used when multiple measurements occurred.

Time to onset was calculated using Kaplan–Meier methodology. The association between OS and CRS incidence was assessed using a multivariate Cox model adjusted for baseline characteristics of age, sex, ECOG, lactate dehydrogenase (LDH), alkaline phosphatase (ALP), largest liver lesion size, and time from diagnosis. In IMCgp100-202, OS was compared between patients receiving systemic corticosteroids within 30 days of the first dose versus no corticosteroids using propensity scores from logistic regression with covariates including age, sex, baseline LDH, ALP, largest metastatic lesion size, ECOG, time from diagnosis, rash occurrence, LFT elevation, and CRS grade ≥2. Covariates were balanced via inverse probability of treatment weighting. Treatment effects were estimated with weighted Kaplan–Meier and Cox models using robust sandwich and bootstrap variance. SAS v9.4 or higher (SAS Institute, Inc.; RRID: SCR_008567) was used for analyses.

## Results

### Patients

A total of 410 patients with mUM were included in the pooled analysis. The median age was 62 years; 75% were ECOG performance status 0, 45% had elevated LDH, and 95% had at least one liver metastasis, with 45% of these patients having at least one liver metastasis >3 cm (M1b-c; Table 1). In the metastatic setting, 61% of patients were treatment naïve. The median duration of tebentafusp treatment was 5.4 months. At the time of data cutoff, half of patients (50%; *n* = 204) had been treated beyond initial radiographic progression by RECIST v1.1 with a median duration of 2.3 months (range, 0.1–28.7+ months), and 23 patients (5.6%) had received tebentafusp for ≥22 months.

**Table 1.** Demographics and baseline characteristics.

Parameter	All studies (*N* = 410)
Median age, years (range)	62 (23–91)
Male, *n* (%)	209 (51%)
ECOG performance status, *n* (%)	
0	309 (75%)
1+	96 (23%)
Missing/not reported	5 (1%)
LDH, *n* (%)	
>ULN	186 (45%)
Missing	3 (1%)
Absolute lymphocyte count, *n* (%)	
<0.5 × 10^9^/L	3 (1%)
<1.0 × 10^9^/L	55 (13%)
≥1.0 × 10^9^/L	355 (87%)
ALP, *n* (%)	
>ULN	102 (25%)
Missing	1 (0.2%)
Stage at initial diagnosis, *n* (%)	
Stage I	65 (16%)
Stage II	133 (32%)
Stage III	96 (23%)
Stage IV	43 (10%)
Median time from initial diagnosis to study treatment, years (min, max)	3.48 (0.1, 28.5)
Median time from metastatic diagnosis to study treatment, years (min, max)	0.32 (0.0, 12.3)
Largest liver lesion diameter (investigator assessment), *n* (%)	
0 to ≤3 cm^a^	205 (50)
>3 cm	186 (45)
No liver lesion	19 (5%)

Abbreviation: ULN, upper limit of normal.

a Non-target liver lesions are counted in the 0 to ≤3 cm category.

### TRAE

The overall safety profile of tebentafusp did not seem to be affected by whether the patients had (IMCgp100-01 and IMCgp100-102) or had not (IMCgp100-202) received any prior therapy for metastatic disease (Supplementary Table S2). Nearly all patients (408 of 410; 99.5%) experienced at least one TRAE, most commonly pyrexia (77%), pruritus (71%), and chills (53%; Table 2). Grade 3 TRAE occurred in 46% (*n* = 188) of patients, with rash maculo-papular (10%), rash (8%), aspartate aminotransferase increased (5%), hypotension (5%), and pruritus (5%) being most frequent. There were only 24 (6%) grade 4 TRAE, with lymphopenia (*n* = 6) and hypotension (*n* = 4) being the most common, and no fatal TRAE among all 410 patients. Very few patients, 2% (*n* = 8), discontinued tebentafusp because of a TRAE (Supplementary Table S2). Dose reductions for AE management were allowed but used infrequently (5%), with most cases (15 of 19) being re-challenges at the same dose during escalation. Likewise, the rate of tebentafusp interruptions (18%) due to a TRAE was also especially low given the weekly dosing regimen. Ninety patients (22%) experienced a treatment-related SAE; the most common were CRS in 29 patients (7%), pyrexia in 15 patients (4%), and hypotension in 11 patients (3%).

**Table 2.** TRAE occurring in patients with mUM treated with tebentafusp.

Preferred term	All studies (*N* = 410)		
	Any grade (≥10%), *n* (%)	Grade 3 (≥1%), *n* (%)	Grade 4 (≥1%), *n* (%)
Any TRAE	408 (99.5%)	188 (46%)	24 (6%)
Pyrexia	317 (77%)	18 (4%)	0
Pruritus	292 (71%)	19 (5%)	0
Chills	218 (53%)	2 (0.5%)	0
Rash	206 (50%)	31 (8%)	0
Nausea	206 (50%)	4 (1%)	0
Fatigue	197 (48%)	14 (3%)	0
Hypotension	167 (41%)	19 (5%)	4 (1%)
Dry skin	137 (33%)	1 (0.2%)	0
Rash maculo-papular	131 (32%)	39 (10%)	0
Vomiting	126 (31%)	3 (1%)	0
Erythema	105 (26%)	4 (1%)	0
Headache	96 (23%)	2 (0.5%)	0
Hair color changes	93 (23%)	1 (0.2%)	0
Edema peripheral	92 (22%)	1 (0.2%)	0
Skin exfoliation	86 (21%)	1 (0.2%)	0
Periorbital edema	80 (20%)	0	0
Aspartate aminotransferase increased	66 (16%)	19 (5%)	2 (0.5%)
CRS^a^	63 (15%)	5 (1%)	0
Abdominal pain	62 (15%)	5 (1%)	0
Alanine aminotransferase increased	60 (15%)	12 (3%)	1 (0.2%)
Diarrhea	54 (13%)	3 (1%)	0
Arthralgia	51 (12%)	2 (0.5%)	0
Skin hypopigmentation	51 (12%)	0	0
Vitiligo	51 (12%)	1 (0.2%)	0
Face edema	50 (12%)	0	0
Decreased appetite	47 (11%)	0	0
Skin hyperpigmentation	45 (11%)	0	0
Lipase increased	43 (10%)	11 (3%)	3 (1%)
Flushing	42 (10%)	0	0
Myalgia	41 (10%)	0	0
Lymphopenia	33 (8%)	10 (2%)	6 (1%)
Hypophosphatemia	31 (8%)	15 (4%)	1 (0.2%)
Dyspnea	27 (7%)	5 (1%)	0
Hyperbilirubinemia	29 (7%)	7 (2%)	0
Rash erythematous	25 (6%)	4 (1%)	0
Rash pruritic	25 (6%)	3 (1%)	0
Hypertension	20 (5%)	13 (3%)	0
Gamma-glutamyltransferase increased	9 (2%)	3 (1%)	1 (0.2%)
Hypoxia	9 (2%)	4 (1%)	0
Syncope	5 (1%)	5 (1%)	0

a Investigator-reported AE.

**Figure 1.**
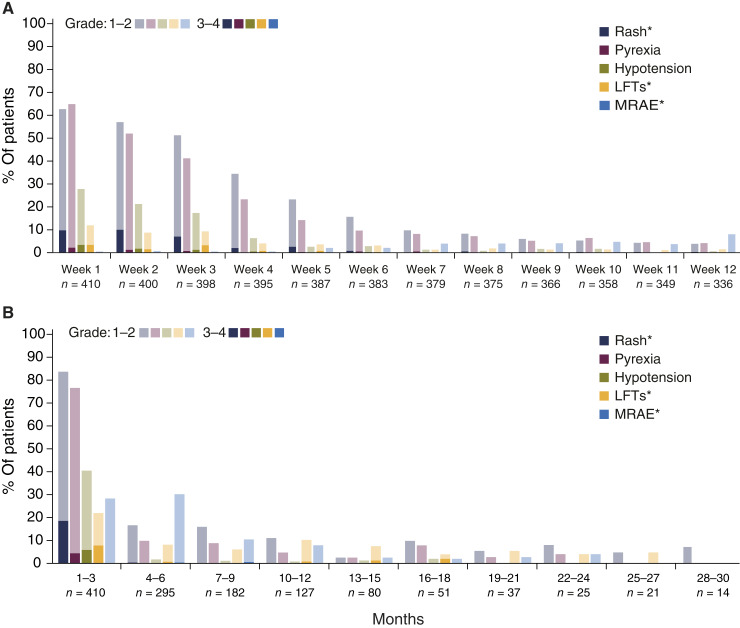
Attenuation of select TRAE with repeated dosing. Incidence and severity of select TRAE over time for patients with mUM treated with tebentafusp across all studies (*N* = 410). **A,** Weekly incidence of TRAE during the acute phase (1–12 weeks). **B,** Long-term incidence of TRAE in 3-month intervals up to 30 months, which can be broken down into acute (months 1–3), intermediate (months 4–12), and late phases (>12 months). *Rash, LFT, and MRAE are composite terms for a list of related AE of any grade (Supplementary Table S1).

Most TRAE, including pyrexia, hypotension, and rash, occurred after the first three to four infusions, with frequency and severity decreasing with repeated dosing (Fig. 1A). These acute TRAE were infrequent following the first 3 months of treatment (acute phase), with other MRAE such as vitiligo and other hypo-/hyperpigmentation events being the most frequently reported TRAE in months 4 to 12 (intermediate phase). Beyond 12 months, the incidence of these events was very low in patients receiving long-term tebentafusp treatment (late phase; Fig. 1B).

The safety profile in patients who received treatment beyond initial radiographic progression was consistent with that expected for patients receiving long-term tebentafusp and none discontinued because of a TRAE (Supplementary Table S3).

### AESI

Consistent with the mechanism of tebentafusp, most TRAE were mediated by cytokines or related to skin. Consequently, CRS per ASTCT criteria, ASR, and elevations in LFT were specified as AESI.

### CRS

**Figure 2.**
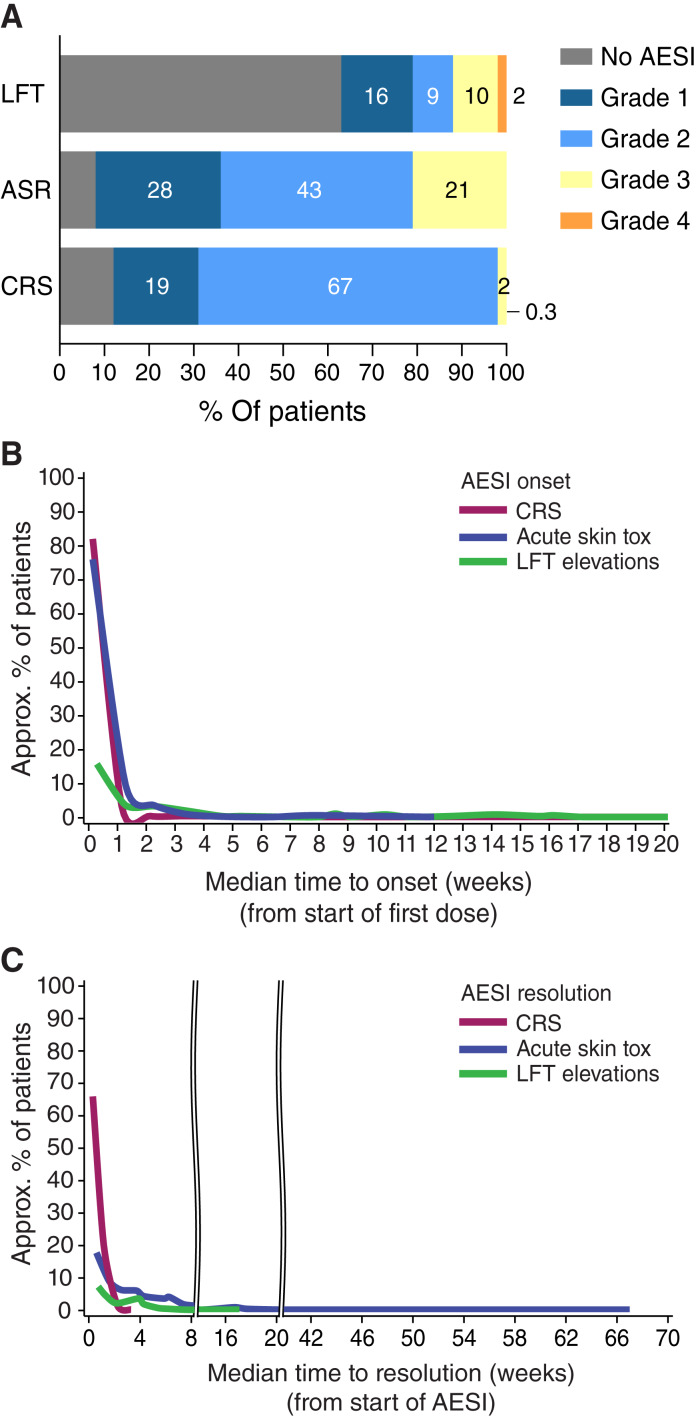
Frequency of AESI by maximum grade and kinetics of onset and resolution of AESI. **A,** Frequency of each AESI by grade. CRS was programmatically graded *post hoc* according to the ASTCT consensus grading criteria for CRS for patients in studies 102 and 202 (*N* = 391). CTCAE grading for treatment-emergent ASR and elevations in LFT for all patients (*N* = 410). **B** and **C,** Median time to (**A**) onset from the start of the first dose or (**B**) resolution from the start of AESI for CRS, ASR, and LFT elevations. The peak of each curve shows the proportion of patients who developed that AESI. CRS was programmatically graded *post hoc* according to the ASTCT consensus grading criteria for CRS for patients in studies 102 and 202 (*N* = 391). CTCAE grading for treatment-emergent ASR and elevations in LFT for all patients (*N* = 410).

To ensure consistency in incidence reporting, CRS was adjudicated *post hoc* for IMCgp100-102 and IMCgp100-202 per the ASTCT 2019 criterion (16). Evaluation and grading of CRS are determined by pyrexia, hypotension, hypoxia, and associated medical interventions. Across both studies, most patients experienced at least one episode of CRS (*n* = 344 of 391; 88%), the majority of which were mild (grade 1, 19%) or moderate (grade 2, 67%) in severity (Fig. 2A; Supplementary Table S4). Most grade 2 CRS events (92%) were due to hypotension, whereas 4.5% of events were attributed to hypoxia and 3.6% involved both conditions. Only a few patients (*n* = 7, 2%) had grade 3 (*n* = 6) or grade 4 (*n* = 1) CRS. There were no instances of immune effector cell-associated neurotoxicity syndrome reported, and no deaths occurred as a result of CRS. Twenty-three patients (6%) had their dose held at least 1 week because of a CRS-related TRAE (e.g., pyrexia, fatigue, and chills) and four patients (1%) discontinued tebentafusp because of CRS. The most frequently reported signs and symptoms of CRS reported were pyrexia, chills, nausea, and hypotension; however, elevated cytokines may also lead to other AE such as fatigue, headache, nausea, and vomiting. Emergent AE that occurred on the same day or the day after incidence of CRS in ≥10% of patients are summarized in Supplementary Table S5.

There were no differences in the incidence of any-grade CRS with respect to age, sex, race, ECOG performance status, baseline values of LDH, ALP, or ALC, or size of the largest liver lesion. However, patients with no liver lesions were at a lower risk of developing CRS (Supplementary Fig. S1A). In phase II, baseline cytokine levels did not correlate with CRS incidence (Supplementary Fig. S2A), but greater peripheral cytokine induction was linked to developing CRS or greater severity (Supplementary Fig. S2C). In terms of efficacy, no significant difference in OS was observed for first-line (1L) or second-line plus (2L+) patients who did not develop CRS or had grade 1 CRS (pyrexia only) versus those who had grade ≥2 CRS after adjusting for baseline covariates [multivariate HR (95% CI), 1.16 (0.58–2.3) for 1L and 1.4 (0.87–2.28) for 2L+].

Most patients experienced CRS after each of the first three tebentafusp infusions, but both incidence and severity decreased with repeated dosing (Supplementary Fig. S3A). In most cases, CRS began within 1 day of infusion (Fig. 2B; Supplementary Table S6). Pyrexia was observed in nearly all CRS cases, with body temperature increasing within 2 to 4 hours and peaking around 8 to 10 hours after tebentafusp infusion (Supplementary Fig. S4A). In grade ≥2 CRS, most patients had decreased blood pressure (BP) within 4 to 6 hours, reaching its maximum drop by 16 hours after infusion (Supplementary Fig. S4B and S4C). Most CRS cases, including those of grade ≥2 severity, resolved by the day after the most recent tebentafusp infusion (Fig. 2C).

#### ASR

Skin TRAE were also very common, affecting 92% of patients, with 21% (*n* = 86) experiencing grade 3 events (Fig. 2A; Supplementary Table S4). Patients with favorable baseline prognostic factors [ECOG 0, liver lesions ≤ 3cm (M1a), and normal LDH and ALP] showed a slightly higher incidence of ASR (Supplementary Fig. S1B). Baseline peripheral cytokine levels were not associated with rash incidence (Supplementary Fig. S2B). In contrast to CRS, cytokine induction was highest in patients without a rash in the first week of treatment and lowest in patients with grade 3 rash (Supplementary Fig. S2D). The most frequently reported ASR were pruritus (71%), rash (50%), rash maculo-papular (32%), erythema (26%), skin exfoliation (21%), and periorbital edema (20%; Supplementary Fig. S5). No cases of Stevens–Johnson syndrome or toxic epidermal necrolysis were reported. No patient discontinued tebentafusp or died from a skin TRAE. The rate of treatment interruptions (2%) and dose reductions (1.2%) due to a skin TRAE was low.

**Figure 3.**
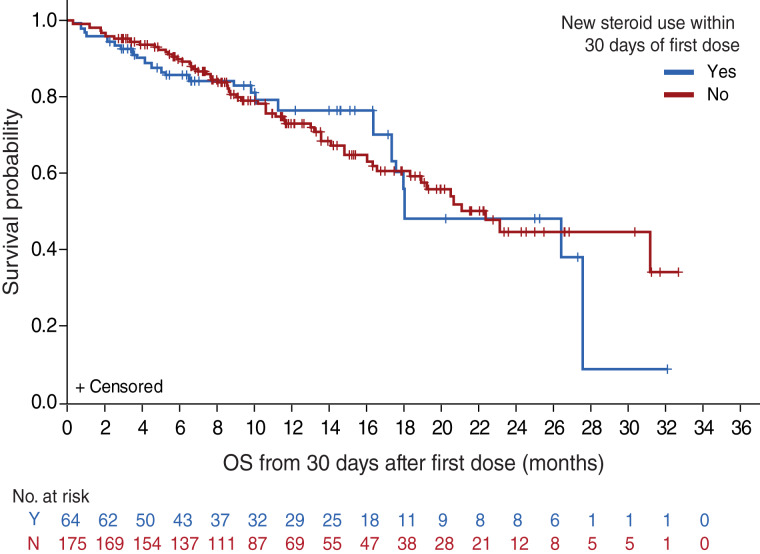
Landmark OS for corticosteroid use within the first 30 days of the first tebentafusp dose. Kaplan–Meier plot of inverse probability of treatment–weighted OS (ATE weights) by corticosteroid use within the first 30 days of the first tebentafusp dose, landmarked at day 30. HR (95% confidence interval), 1.09 (0.67–1.76). ATE, average treatment effects.

As seen for CRS and related symptoms, most skin TRAE (e.g., rash, pruritus, and edema) occurred during the initial three tebentafusp doses (Fig. 1; Supplementary Fig. S3B). The onset of acute skin TRAE usually occurred within 1 day of dosing (Fig. 2B). Although the median time to resolution for any grade skin TRAE was 31 days (Fig. 2C), the majority of grade ≥2 skin TRAE improved to grade ≤1 within 7 days, allowing patients to continue weekly treatment. By contrast, the less frequent events of hypo-/hyperpigmentation (collectively termed MRAEs; Supplementary Fig. S5), occurring in 48% of patients, had a later median onset of 80 days and typically improved or resolved following tebentafusp discontinuation (median time to resolution, 195 days; Figs. 1 and 3). No patient had their treatment discontinued or interrupted because of an MRAE.

#### LFT abnormalities

Because of the prevalence of liver metastases among patients with mUM and the potential for inflammatory cytokines to elevate LFT, hepatic AE were closely monitored during clinical development, with laboratory data used to summarize LFT kinetics and outcomes for patients. During the initial 3 weeks of tebentafusp treatment, 40% (*n* = 165) and 20% (*n* = 84) of patients had at least one increase of CTCAE grade ≥1 in laboratory measurements of aminotransferases or bilirubin, respectively. However, most of these elevations were not considered clinically relevant as they were generally mild and resolved without intervention and did not meet the criteria for AE.

Elevated LFT-emergent AE of any grade were reported in 37% of patients (Fig. 2A), with treatment-related LFT elevations occurring in 26% of patients, and 8% of patients had a grade 3 or 4 TRAE (Supplementary Table S4). These events mostly occurred during the first 3 weeks of treatment and tapered over time (Fig. 1). The median time to onset was 13.5 days and the median time to resolution was 22 days (Fig. 2C).

LFT elevations occurring after dose escalation were generally not linked with other mechanism-related AE like CRS or rash and often coincided with disease progression. One patient discontinued tebentafusp because of an LFT elevation, but no hepatic TRAE was associated with a patient death (Supplementary Table S4).

In a subset analysis, patients with a liver lesion >3 cm or elevated baseline values for LDH or ALP were at higher risk of developing elevated LFT during treatment (Supplementary Fig. S1C). Given that these baseline characteristics are known poor prognostic indicators in mUM, it is possible that LFT elevations in these patients may be due to disease progression of their liver lesions. Conversely, in other patients, transient elevations are more likely to be a result of a drug effect.

#### Eye AE

Eye disorders were not classified as an AESI, as they were less frequent but were initially identified as a risk since gp100-positive intraocular melanocytes could be targeted by tebentafusp. Thorough ophthalmologic exams conducted in the first-in-human study (IMCgp100-01) did not reveal any concerning or related intraocular clinical findings or overt vision changes. Across all studies, 42% of patients had an emergent ocular AE. Only four patients had a grade 3 or 4 event, and two were treatment related. Most reported ocular AE were extraocular and due to inflammation or periorbital skin AE, including periorbital edema (80 patients, 20%), eye pain (17 patients, 4%), and ocular pruritus (10 patients, 2%; Supplementary Table S7). As these ocular AE involve melanocytes in the skin around the eye, they are included in the composite AESI term for ASR (Supplementary Fig. S5 and Supplementary Table S1).

### Management of AESI

Following observations from IMCgp100-01, the protocols for IMCgp100-102 and IMCgp100-202 mandated a 16-hour monitoring period after each of the first three tebentafusp infusions to enable sufficient time to detect and promptly manage initial symptoms of CRS. Together with the protocol-recommended AE management guidelines (1, 11), this management algorithm resulted in a low rate of treatment-related discontinuation and no treatment-related deaths. Retrospective analysis of key vital signs (temperature and BP) found that temperature increase seemed to be among the first signs of CRS, with affected patients presenting with an increase in temperature of at least 1°C from pre-dose baseline at 4 to 6 hours and peaking at 8 to 10 hours, from the start of tebentafusp treatment (Supplementary Fig. S4A). Both systolic and diastolic BP decreased within the first 12 hours for both grade 1 and grade ≥2 CRS (Supplementary Fig. S4B and S4C).

Unlike other trials of T-cell engagers, none of the three tebentafusp trials mandated any premedications as primary prophylaxis for CRS. The medications most frequently used to treat CRS were antipyretics (88%), intravenous fluids (45%), and systemic corticosteroids (25%; Table 3); four (1%) patients received tocilizumab. Most patients with CRS (64%) received only a single dose of systemic corticosteroid to manage their CRS episodes, and in a *post hoc* propensity score analysis of IMCgp100-202, early corticosteroid use to manage AE did not negatively affect OS when adjusted for baseline prognostic factors (Fig. 3).

**Table 3.** Medications used to treat AESI.

Treatment group	Medication received	Grade 1, *n* (%)	Grade 2, *n* (%)	Grade 3, *n* (%)	Grade 4, *n* (%)	Total *n* (%)
Patients experiencing CRS (*n* = 344)	Antipyretics	161 (47%)	220 (64%)	4 (1%)	1 (0.3%)	304 (88%)
	IV fluids	4 (1%)	151 (44%)	3 (1%)	0	155 (45%)
	Corticosteroids	24 (7%)	67 (19%)	4 (1%)	1 (0.3%)	86 (25%)
	Oxygen	0	26 (8%)	5 (1.5%)	1 (0.3%)	32 (9%)
	Vasopressors	0	1 (0.3%)^a^	2 (0.6%)	1 (0.3%)	4 (1%)
	Tocilizumab	1 (0.3%)	2 (0.6%)	0	1 (0.3%)	4 (1%)
Patients experiencing ASR (*n* = 361)	Antihistamines	216 (60%)	166 (46%)	55 (15%)	—	276 (76%)
	Dermatologic or topical corticosteroid	153 (42%)	114 (32%)	38 (11%)	—	212 (59%)
	Any systemic corticosteroid	17 (5%)	26 (7%)	8 (2%)	—	34 (9%)
	Systemic IV corticosteroid	11 (3%)	17 (5%)	7 (2%)	—	25 (7%)
	Systemic oral corticosteroid	4 (1%)	11 (3%)	2 (0.5%)	—	12 (3%)
Patients experiencing LFT elevations (*n* = 146)	Any systemic corticosteroid	8 (5%)	13 (9%)	11 (8%)	1 (0.7%)	15 (10%)
	Systemic IV corticosteroid	6 (4%)	9 (6%)	6 (4%)	1 (0.7%)	10 (7%)
	Systemic oral corticosteroid	4 (3%)	7 (5%)	7 (5%)	0	10 (7%)

Abbreviation: IV, intravenous.

a Patient in study 202 had intramuscular epinephrine listed as a concomitant medication for contrast allergy and not for CRS, which was never used. After the data cutoff, the entry was removed from the site’s database.

Many skin symptoms resolved without systemic corticosteroid (Table 3); in the few instances systemic corticosteroids were used, only short-term (median, 1 day) systemic corticosteroid treatment was necessary. Antihistamines were most frequently used to manage skin symptoms (76%), followed by topical corticosteroids (59%), with hydrocortisone, clobetasol, or betamethasone being most prescribed.

Overall, most changes in LFT were generally mild to moderate in severity and required no treatment. A few patients with a hepatic TRAE received at least one concomitant medication (*n* = 26; 16 received corticosteroids; Table 3) or dose modification (*n* = 5).

In a retrospective analysis of hospitalizations, defined as either an extension of the mandated monitoring period during intra-patient dose escalation or an unplanned hospitalization at any time, in IMCgp100-202, a similar percentage of patients experienced an SAE-related hospitalization in the tebentafusp arm (24%) as in the investigator’s choice control arm (23%; Supplementary Table S8). In the tebentafusp group, most (65%) SAE-related hospitalizations occurred during the intra-patient dose-escalation period. After reaching the maintenance dose of 68 mcg of tebentafusp, 24 patients (10%) had an unplanned hospitalization due to an SAE, 12 (5%) experienced a treatment-related SAE, and 44% of these visits occurred before week 9. The majority of SAE-related hospitalizations in the tebentafusp arm at any time were due to CRS (17/88; 19%), rash (6/88; 7%), pyrexia (6/88; 7%), and hypotension (6/88; 7%). Overall, the frequency of hospitalizations due to grade ≥3 SAE was lower in the tebentafusp arm (54%) when compared with those who received the investigator’s choice of therapy (92%).

## Discussion

mUM is a life-threatening disease with a poor prognosis. Tebentafusp is the first therapy to improve OS in HLA-A*02:01–positive patients with mUM and the first TCR therapy to receive marketing authorization (1, 17, 18). Across 410 patients in three clinical trials, tebentafusp was well tolerated, with few treatment-related discontinuations and no treatment-related deaths. The most frequent TRAE, CRS and skin AE, were consistent with the mechanism of action, occurred mainly after the initial three infusions, and were reversible and manageable with appropriate surveillance and intervention. Fewer than one-fifth of patients had their treatment interrupted to manage an AE, with most treatment interruptions occurring for reasons unrelated to treatment (e.g., holiday). Most interruptions were short (one or two missed doses) and, in a separate *post hoc* analysis, were shown not to affect outcomes (19). Dose reductions were allowed but used infrequently to manage AE and are therefore not recommended in the post-marketing environment.

CRS is a well-known AE associated with rapid T-cell activation from all T-cell engagers (16). Although investigator-reported CRS was only 15%, *post hoc* grading per ASTCT criteria (16), which are based on the presence of pyrexia, hypotension and hypoxia, and related interventions, showed that 88% of patients experienced CRS. However, the incidence of severe CRS (grade 3–4) was low at 2%, which is notable since, unlike other T-cell engagers, prophylactic corticosteroids were not mandated or frequently used. Unlike chimeric antigen receptor T (CAR-T)–associated CRS, tebentafusp-related CRS was predictable in onset and resolution, did not include neurologic sequelae (i.e., no immune effector cell-associated neurotoxicity syndrome), and was usually managed with antipyretics and intravenous fluids, with supplemental oxygen or short courses of intravenous corticosteroids (majority received a single dose) when needed; anti–IL-6 (i.e., tocilizumab) was rarely used. Short-term corticosteroids (1–2 days) for AE management did not appear to compromise efficacy. Intravenous fluids with timely antipyretic usage for the first 3 weekly treatments with dose escalation typically prevent worsening of CRS symptoms. If a patient with CRS does not improve with fluids, antipyretics, or oxygen, the use of corticosteroids does not seem to negatively affect outcomes. Future studies will explore whether prophylactic corticosteroids can further improve tolerability without affecting efficacy.

Because CRS can escalate if symptoms are not recognized and appropriate management is not instituted promptly, a 16-hour observation period was selected as any cases of hypotension would likely occur in this window. After the third dose, CRS incidence and severity decrease such that the 16-hour observation is typically unnecessary beyond this point. An early temperature increase (≥1°C) or blood pressure drop within 8 to 10 hours may be an early indicator of CRS. Once CRS is detected, patients should be treated per the labeled or local guidelines until symptom resolution. Premedication may be necessary before future doses for patients who experienced moderate-to-severe (grade ≥2) prior CRS episodes. Although only a small number of patients with adrenal insufficiency were studied during tebentafusp development, both clinical and post-marketing experience support the potential for more severe and prolonged CRS-related hypotension. Thus, we consider it clinically prudent that patients with preexisting adrenal insufficiency who are on replacement systemic corticosteroids receive stress dose steroids before and after the first four to five tebentafusp administrations to minimize the risk of observing severe or persistent hypotension associated with CRS.

In these international studies, some patients were hydrated with intravenous fluids before tebentafusp infusion to prevent CRS-related hypotension. Hence, it is clinically appropriate to assess the volume status of patients and consider intravenous fluids as necessary. Likewise, because of the anticipated risk of hypotension, patients with preexisting hypertension were required to temporarily suspend antihypertensive medications prior to the first few doses of tebentafusp during development. However, this is no longer recommended in the postapproval setting because (i) BP reductions were similar regardless of antihypertensive use; (ii) pausing antihypertensive medication likely contributed to the increased frequency of hypertension AE reported among patients receiving tebentafusp versus investigator’s choice in the phase III study (1, 18); and (iii) pausing antihypertensive medication in patients with a known cardiac history could exacerbate the risk for sequelae in the context of CRS.

Skin TRAE were common and decreased with repeated dosing. Skin biopsies, including histology, gene expression profiling, and single-cell analysis, have clearly shown that tebentafusp redirects T cells (mainly CD8^+^) to gp100-positive melanocytes in the skin (15, 20). Symptoms can be distressing for patients who should therefore be forewarned/prepared for this very common AE. Management should be tailored appropriately to the severity and area affected (e.g., face, genital area, and extremities). In most cases, antihistamines or topical corticosteroid treatments such as hydrocortisone, clobetasol, or betamethasone were found to be sufficient to ameliorate symptoms, and the majority of skin TRAE will improve within a few days to a week.

Neither rash nor CRS, a monocyte-driven event, was independently associated with clinical benefit. Although a rash in week one was initially observed to correlate with outcomes in the phase I/II study, leading to its inclusion as a co-primary endpoint in the phase III study, there was no significant association between rash and OS observed in the latter after adjusting for baseline covariates (15, 21). Interestingly, analysis of skin biopsies from tebentafusp-treated patients found that the development of rash depends on baseline expression of gp100 and other melanin pathway genes in the skin, as well as T-cell infiltration into the skin (15, 20). Thus, whether a patient develops a rash early on-treatment may depend on increased T-cell infiltration and higher gp100 engagement in the skin or on a chronic inflammatory state in advanced disease that delays the immune response. A recent retrospective analysis using samples from two ImmTAC trials (IMCgp100-102 and IMC-F106C-101) showed that patients with higher T-cell fitness, defined based on a three-gene expression signature in blood that is associated with naïve/stem memory T cells, had more MRAE, aligning with T-cell redirection to gp100-expressing melanocytes. No association with CRS, driven by monocyte activation, was found (22). In keeping with this, we observe here that greater peripheral cytokine induction after tebentafusp infusion was seen in patients without rash or with severe CRS, again indicating mechanistic differences between a T-cell–driven rash and monocyte-driven CRS.

Due to the location of primary disease and expression of gp100 by intraocular melanocytes, ocular AE were also closely monitored in these studies. However, nearly all reported ophthalmologic AE were of CTCAE grade 1 or 2, did not affect vision, and were consistent with periocular skin involvement rather than intraocular toxicity.

Because most patients with mUM have liver metastases and tebentafusp induces inflammation and cytokines in these lesions (10, 12), elevated aminotransferases and, to a lesser extent, bilirubin were common in the initial weeks of treatment. These elevations were generally mild laboratory observations, not requiring treatment or tebentafusp discontinuation, and decreased in frequency over time. Hepatocytes do not express gp100 and tebentafusp did not redirect T cells against normal hepatocytes in preclinical *in vitro* studies. Early (first 6 weeks) increases in LFT on tebentafusp should be expected and are consistent with the mechanism of action; however, later in the treatment course, patients should be monitored for onset of liver injury because of disease progression, as tumor progression within the liver can result in liver injury with an increase in LFTs (23).

In summary, tebentafusp is the first therapy to show an OS benefit in HLA-A*02:01–positive mUM and the first TCR-based therapy to receive regulatory authorization. The majority of tebentafusp TRAE are consistent with T-cell activation (e.g., CRS) or redirection of T cells to the skin (e.g., rash and pruritus). These TRAE occur shortly after the initial doses and decrease in frequency and severity with repeated dosing. Close observation for CRS is critical during these initial doses to promptly treat and prevent escalation. With the monitoring and management algorithm, tebentafusp had a very low rate of treatment-related discontinuation and no treatment-related deaths. Importantly, the safety profile did not change with extended follow-up in the phase II and III trials (18, 24), and there has been no incidence of the autoimmune immune related adverse events that are commonly observed with immune checkpoint inhibitor treatments.

## Supplementary Material

Supplementary Data 1 Contains all supplementary tables and figures

## Data Availability

Access to summary outputs (tables or figures) of the data underlying this article may be granted to qualified academic researchers in the field upon request and approval by the study management committee and subject to appropriate data sharing and transfer agreements. Requesters should submit a proposal including purpose, data format (e.g., SAS files), hypothesis, and specific rationale to info@immunocore.com.
