# Supplementary material for: Incidence and Management of Adverse Events Associated with Tebentafusp Treatment in Metastatic Uveal Melanoma: Pooled Safety Analysis of 410 Patients
Source: Clin Cancer Res. 2025 Sep 12;31(23):5027–36. doi: 10.1158/1078-0432.CCR-25-1513 (PMC12666308; doi:10.1158/1078-0432.CCR-25-1513)
Supplement: Supplementary Data 1 — Contains all supplementary tables and figures [file ccr-25-1513_supplementary_data_1_suppds1.pdf]

## Supplementary Appendix

This appendix is a supplement to: Takami Sato, Marcus O. Butler, Sophie Piperno-Neumann, et al. Incidence and Management of Adverse Events Associated with Tebentafusp Treatment in Metastatic Uveal Melanoma: Pooled Safety Analysis of 410 patients

## Contents


Supplementary Table S1. Listing of preferred terms included in the composite terms 2

Supplementary Table 2. Summary of treatment-related adverse events by study 3

Supplementary Table 3. All Grade TRAEs occurring in ≥ 5% of 1L patients treated beyond initial radiographic progression in study IMCgp100-202 4

Supplementary Table 4. Summary of AESIs 5

Supplementary Table 5. Emergent AEs occurring concurrent with CRS in ≥ 5% of patients 6

Supplementary Table 6. Time to onset and resolution 7

Supplementary Table 7. Summary of eye AEs occurring in ≥1% of patients 8

Supplementary Table 8. Summary of patients with extended or unplanned hospitalizations due to SAEs in study IMCgp100-202[a] 9

Supplementary Table 9. Representativeness of Study Participants 10

Supplementary Figure 1. Subgroup analyses of AESIs 12

Supplementary Figure 2. Cytokine induction in patients experiencing CRS or rash 15

Supplementary Figure 3. Incidence of AESIs over time 16

Supplementary Figure 4. Mean change in body vital signs associated with CRS 17

Supplementary Figure 5. Acute skin reaction TRAEs and MRAEs 18

**Supplementary Table S1.** Listing of preferred terms included in the composite terms

| Toxicity group | | Preferred Terms (MedDRA v23.1) |
|---|---|---|
| **Acute skin reactions** | Rash | Blister, Dermatitis, Dermatitis acneiform, Dermatitis allergic, Dermatitis bullous, Dermatitis contact, Dermatosis, Drug eruption, Eczema, Eczema eyelids, Erythema multiforme, Exfoliative rash, Interstitial granulomatous dermatitis, Lichenification, Lichenoid keratosis, Palmar-plantar erythrodysesthesia syndrome, Papule, Psoriasis, Rash, Rash erythematous, Rash macular, Rash maculo-papular, Rash papular, Rash pruritic, Rash vesicular, Seborrhoea, Seborrheic dermatitis, Skin abrasion, Skin erosion, Skin exfoliation, Skin irritation, Skin plaque, Solar dermatitis, Urticaria |
| | Pruritus | Eye pruritus, Eyelids pruritus, Pain of skin, Pruritus, Sensitive skin, Skin burning sensation, Urticaria chronic |
| | Erythema | Conjunctival hyperaemia, Erythema, Erythema of eyelid, Eye inflammation, Ocular hyperaemia, Photosensitivity reaction |
| | Edema | Circumoral swelling, Conjunctival oedema, Eye oedema, Eye swelling, Eyelid oedema, Periorbital oedema, Skin induration, Skin tightness, Swelling of face, Swelling of eyelid |
| **Melanocyte related AE (MRAE)** | | Achromotrichia acquired, Ephelides, Eyelash discolouration, Eyelash hypopigmentation, Hair colour changes, Lentigo, Pigmentation disorder, Skin depigmentation, Skin discolouration, Skin hyperpigmentation, Skin hypopigmentation, Solar lentigo, Vitiligo |
| **LFT elevations** | Narrow terms | Alanine aminotransferase abnormal, Alanine aminotransferase increased, Ascites, Aspartate aminotransferase increased, Autoimmune hepatitis, Bilirubin urine present, Blood bilirubin increased, Blood fibrinogen decreased, Cholestasis, Gamma-glutamyltransferase increased, Hepatic enzyme increased, Hepatic failure, Hepatic necrosis, Hepatic pain, Hepatocellular injury, Hepatomegaly, Hepatotoxicity, Hyperammonaemia, Hyperbilirubinaemia, Hypertransaminasaemia, Immune-mediated hepatitis, International normalised ratio increased, Jaundice, Jaundice cholestatic, Liver disorder, Liver function test abnormal, Liver function test increased, Ocular icterus, Prothrombin level decreased, Prothrombin time prolonged, Steatohepatitis, Transaminases increased, Urine bilirubin increased, Varices oesophageal |

**Supplementary Table 2.** Summary of treatment-related adverse events by study

| | IMCgp100-01 (N=19) n (%) | IMCgp100-102 (N=146) n (%) | IMCgp100-202 (N=245) n (%) | All Studies (N = 410) n (%) |
|---|---|---|---|---|
| Any grade TRAE | 19 (100%) | 146 (100%) | 243 (99%) | 408 (99.5%) |
| Grade 3-4 TRAE | 12 (63%) | 74 (51%) | 109 (44%) | 195 (48%) |
| Any treatment-related SAE | 3 (16%) | 33 (23%) | 54 (22%) | 90 (22%) |
| Any TRAE leading to permanent discontinuation of tebentafusp | 0 | 3 (2%) | 5 (2%) | 8 (2%) |
| Any TRAE leading to drug interruptions of tebentafusp | 1 (5%) | 27 (18%) | 44 (18%) | 72 (18%) |
| Any TRAE leading to dose reductions of tebentafusp | 0 | 7 (5%) | 12 (5%) | 19 (5%) |
| A TRAE leading to death | 0 | 0 | 0 | 0 |

TRAE, treatment-related adverse event; SAE, serious adverse event

**Supplementary Table 3.** All Grade TRAEs occurring in ≥ 5% of 1L patients treated beyond initial radiographic progression in study IMCgp100-202

| Preferred Term | Tebe Post RECIST-PD (N=109) | | Tebe Overall (N=245) | |
|---|---|---|---|---|
| | Any Grade | ≥ Grade 3 | Any Grade | ≥ Grade 3 |
| Pyrexia | 15 (14%) | 0 | 185 (76) | 9 (4%) |
| Pruritus | 12 (11%) | 0 | 169 (69%) | 11 (5%) |
| Fatigue | 12 (11%) | 0 | 101 (41%) | 7 (3%) |
| Nausea | 11 (10%) | 0 | 105 (43%) | 2 (1%) |
| Chills | 10 (9%) | 1 (1%) | 114 (47%) | 1 (0.4%) |
| Lipase increased | 10 (9%) | 0 | 32 (13%) | 9 (4%) |
| Hair colour changes | 9 (8%) | 1 (1%) | 48 (20%) | 1 (0.4%) |
| Rash maculo-papular | 8 (7%) | 0 | 74 (30%) | 21 (9%) |
| Vitiligo | 8 (7%) | 0 | 40 (16%) | 0 |
| Vomiting | 7(6%) | 0 | 64 (26%) | 1 (0.4%) |
| Aspartate aminotransferase increased | 7 (6%) | 0 | 47 (19%) | 11 (5%) |
| Rash | 7 (6%) | 0 | 135 (55%) | 23 (9%) |
| Decreased appetite | 6 (6%) | 0 | 30 (12%) | 0 |
| Skin hyperpigmentation | 6 (6%) | 0 | 19 (8%) | 0 |
| Diarrhoea | 5 (5%) | 0 | 31 (13%) | 2 (1%) |
| Alanine aminotransferase increased | 5 (5%) | 0 | 43 (18%) | 7 (3%) |
| Blood alkaline phosphatase increased | 5 (5%) | 0 | 15 (6%) | 1 (0.4%) |
| Erythema | 5 (5%) | 0 | 56 (23%) | 0 |

**Supplementary Table 4.** Summary of AESIs

| Patients with | CRS per ASTCT (N = 391) | Acute skin reactions (N=410) | LFT elevations (N=410) |
|---|---|---|---|
| Any TEAE | 344 (88%) | 379 (92%) | 152 (37%) |
| Any TRAE | 344 (88%) | 377 (92%) | 107 (26%) |
| Any Grade 3 or 4 TRAE | 7 (2%) | 86 (21%) | 33 (8%) |
| Any serious TRAE | - | 18 (4%) | 10 (2%) |
| Any TRAE leading to drug interruptions of tebentafusp | - | 8 (2%) | 14 (3%) |
| Any TRAE leading to dose reductions of tebentafusp | - | 5 (1%) | 5 (1%) |
| A TRAE leading to permanent discontinuation of tebentafusp | 4 (1%) | 0 | 1 (0.2%) |
| A TRAE leading to death | 0 | 0 | 0 |

AESI, adverse event of special interest; ASTCT, American Society for Transplantation and Cellular Therapy; CRS, cytokine release syndrome; LFT, liver function tests; TEAE, treatment-emergent AE; TRAE, treatment-related adverse event

Patients with multiple events in the same category are counted only once in that category. Patients with events in more than one category are counted once in each of those categories.

**Supplementary Table 5.** Emergent AEs occurring concurrent with CRS in ≥ 5% of patients

| **MedDRA**<br><br>**Preferred Term** | **All Studies (N = 391)** [a]<br>**n (%)** |
|---|---|
| Any emergent AE on same day or day after first CRS event | 336 (86%) |
| Pyrexia | 272 (70%) |
| Chills | 130 (33%) |
| Hypotension | 104 (27%) |
| Pruritus | 100 (26%) |
| Rash | 96 (25%) |
| Nausea | 85 (22%) |
| Vomiting | 58 (15%) |
| Fatigue | 56 (14%) |
| Rash maculo-papular | 53 (14%) |
| Cytokine release syndrome[b] | 49 (13%) |
| Erythema | 46 (12%) |
| Periorbital oedema | 41 (11%) |
| Headache | 39 (10%) |
| Tachycardia | 28 (7%) |
| Oedema peripheral | 28 (7%) |
| Aspartate aminotransferase increased | 27 (7%) |
| Face oedema | 25 (6%) |
| Alanine aminotransferase increased | 20 (5%) |
| Dry skin | 20 (5%) |

[a] Includes only Studies 102 and 202; patients with multiple AEs per SOC or PT are counted only once in each row

[b] investigator reported cytokine release syndrome

CRS, cytokine release syndrome

**Supplementary Table 6.** Time to onset and resolution

| Preferred Term / AESI / Composite term | Median Time to onset, days (min, max) | Median Time to resolution, days (min, max) | Time to resolution 90% of patients, days |
|---|---|---|---|
| Acute skin toxicities | 1 (1, 84) | 31 (1, 1340) | 274 |
| Cytokine release syndrome | 1 (1, 225) | 2 (1, 198) | 6 |
| Pyrexia | 1 (1, 914) | 2 (1, 1031) | 8 |
| Hypotension | 2 (1, 340) | 2 (1, 764) | 5 |
| LFT elevations | 13.5 (1, 1412) | 22 (1, 730) | 100 |
| MRAE | 80 (1, 394) | 195 (1, 1378) | 575 |

LFT, liver function tests; MRAE, melanocyte-related adverse events

**Supplementary Table 7.** Summary of eye AEs occurring in ≥1% of patients

| | TEAEs (≥1%) | | TRAEs | |
|---|---|---|---|---|
| **System Organ Class / Preferred Term** | **Any Grade n (%)** | **Grade 3-4 n (%)** | **Any Grade n (%)** | **Grade 3-4 n (%)** |
| Eye disorders | 173 (42) | 4 (1) | 135 (33) | 2 (0.5) |
| Periorbital oedema | 80 (20) | 0 | 80 (20) | 0 |
| Dry eye | 18 (4) | 0 | 8 (2) | 0 |
| Vision blurred | 18 (4) | 0 | 6 (2) | 0 |
| Eye pain | 17 (4) | 1 (0.2) | 6 (2) | 1 (0.2) |
| Lacrimation increased | 14 (3) | 0 | 5 (1) | 0 |
| Eye pruritus | 10 (2) | 0 | 10 (2) | 0 |
| Visual impairment | 10 (2) | 0 | 3 (0.7) | 0 |
| Ocular hyperaemia | 9 (2) | 0 | 3 (0.7) | 0 |
| Eyelash discolouration | 8 (2) | 0 | 8 (2) | 0 |
| Eye oedema | 7 (2) | 0 | 4 (1) | 0 |
| Eyelid oedema | 7 (2) | 0 | 7 (2) | 0 |
| Vitreous floaters | 7 (2) | 0 | 4 (1) | 0 |
| Cataract | 6 (1) | 2 (0.5) | 1 (0.2) | 1 (0.2) |
| Eye swelling | 6 (1) | 0 | 5 (1) | 0 |
| Eye irritation | 5 (1) | 0 | 2 (0.5) | 0 |
| Swelling of eyelid | 5 (1) | 0 | 3 (0.7) | 0 |
| Eye discharge | 4 (1) | 0 | 1 (0.2) | 0 |
| Uveitis | 4 (1) | 0 | 4 (1) | 0 |

**Supplementary Table 8.** Summary of patients with extended or unplanned hospitalizations due to SAEs in study IMCgp100-202[a]

| | Tebentafusp | | | |
|---|---|---|---|---|
| | **Any time (N=245)** | **Intra-patient escalation (IE)[b] (N=245)** | **post-IE[b] (N=245)** | **Investigator's choice (N=111)** |
| Any SAE | 60 (24%) | 44 (18%) | 24 (10%) | 25 (23%) |
| Treatment-related SAE | 46 (19%) | 40 (16%) | 12 (5%) | 8 (7%) |
| Any G3+ SAE | 35 (14%) | 23 (9%) | 14 (6%) | 23 (21%) |
| Treatment related G3+ SAE | 22 (9%) | 19 (8%) | 4 (2%) | 8 (7%) |
| SAEs leading to treatment discontinuation | 6 (2%) | 4 (2%) | 2 (1%) | 5 (5%) |
| SAEs leading to death | 0 | 0 | 0 | 2 (2%) |
| Median duration of hospitalization | 2 | 2 | 4.5 | 5 |

[a] Patients with at least one documented hospitalization where the reason stated is due to an SAE
[b] Patients can be counted in both the intra-patient escalation (IE) and post-IE period

**Supplementary Table 9.** Representativeness of Study Participants

| Cancer type(s)/ subtype(s)/ stage(s)/condition | Uveal melanoma |
|---|---|
| Considerations related to: | |
| Sex | The distribution is roughly equal between men and women at diagnosis of primary uveal melanoma, though some registries show a slight male predominance in metastatic disease cohorts: 52% male vs. 48% female. |
| Age | Median age at diagnosis of primary disease is ~62 yrs. The median age at diagnosis of metastatic disease is typically around 55–65 years. Metastases can develop months to decades after the initial ocular diagnosis, but most occur within 2–5 years. |
| Race/ethnicity | Uveal melanoma is overwhelmingly a disease of people of European (Caucasian) ancestry, accounting for 95–98% of cases. Incidence in non-White populations is quite low: Asian and Hispanic <2% combined; Black patients ~0.6% |
| Geography | Highest incidence is in Northern Europe and North America, reflecting the racial composition of these regions. |

| Cancer type(s)/ subtype(s)/ stage(s)/condition | Uveal melanoma |
|---|---|
| Other considerations | Nearly all published clinical trial participants for uveal melanoma (UM), including the trials of tebentafusp reported herein, have been White/European ancestry patients, reflecting the overwhelming incidence pattern. Black, Asian, and Hispanic patients are virtually absent from pivotal studies resulting in underrepresentation. Registry reviews suggest that Black and Hispanic patients are more likely to present at advanced stages, less likely to undergo eye-preserving therapies, and may experience worse survival. However, these findings are somewhat limited by very small sample sizes.<br>In the USA, access to dedicated ocular oncology centers can be uneven, and patients in rural locations or without insurance may face delayed diagnosis and reduced access to trials. Patients in northern Europe (where incidence is higher) benefit from established referral pathways (e.g., Liverpool Ocular Oncology Centre in the UK, Leiden in the Netherlands). In southern Europe, incidence is lower, but this may also limit local expertise and trial site availability. As trial infrastructure is concentrated in North America and northern/central Europe, access to novel therapies in the Global South are extremely limited. This introduces clear geographic inequities: patients in low-incidence regions who do develop UM have little or no access to trials and are reliant on expanded access programs or off-label systemic melanoma regimens. |
| Overall representativeness of this study | All patients (N=410) were from North America (49%) or Europe (51%). Most patients (92%) were White; Race was not reported, not allowed per local regulation, indicated as 'other' or unknown in 7% of patients. The median age was 62 years (range: 23 to 91 years); 57% of patients were aged under 65 years, 33% were aged between 65 and 75 years, and 9% were aged 75 years or older. Slightly more males were enrolled than females (51% vs. 49%). |

**Supplementary Figure 1.** Subgroup analyses of AESIs

**A**

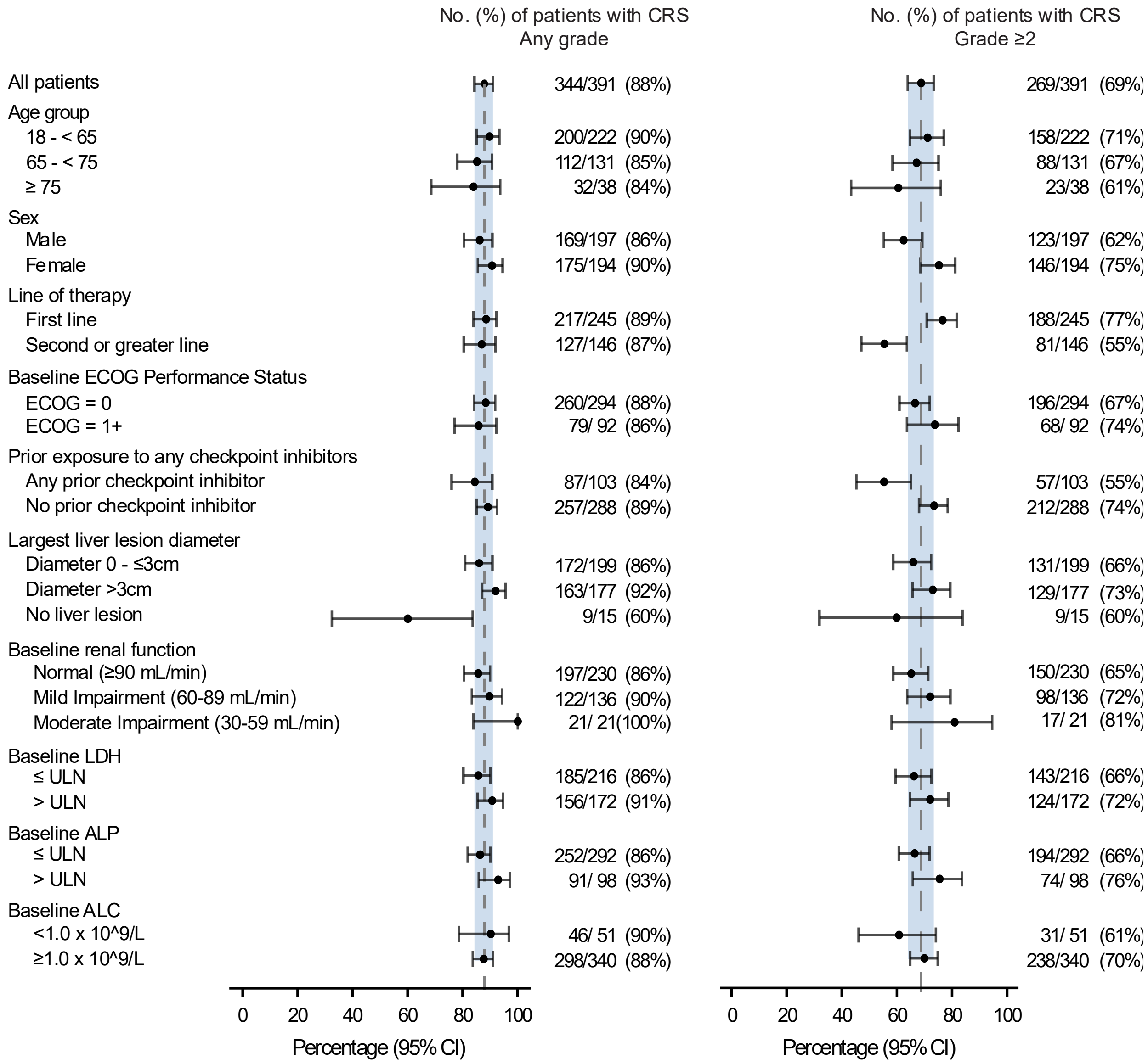


**Supplementary Figure 1. Subgroup analyses of AESIs. (A)** Overall incidence of CRS (any grade and grade ≥2) by disease characteristic subgroup (N=391). Abbreviations: AESI, adverse event of special interest; CI, confidence interval; ULN, upper limit of normal.

**B**

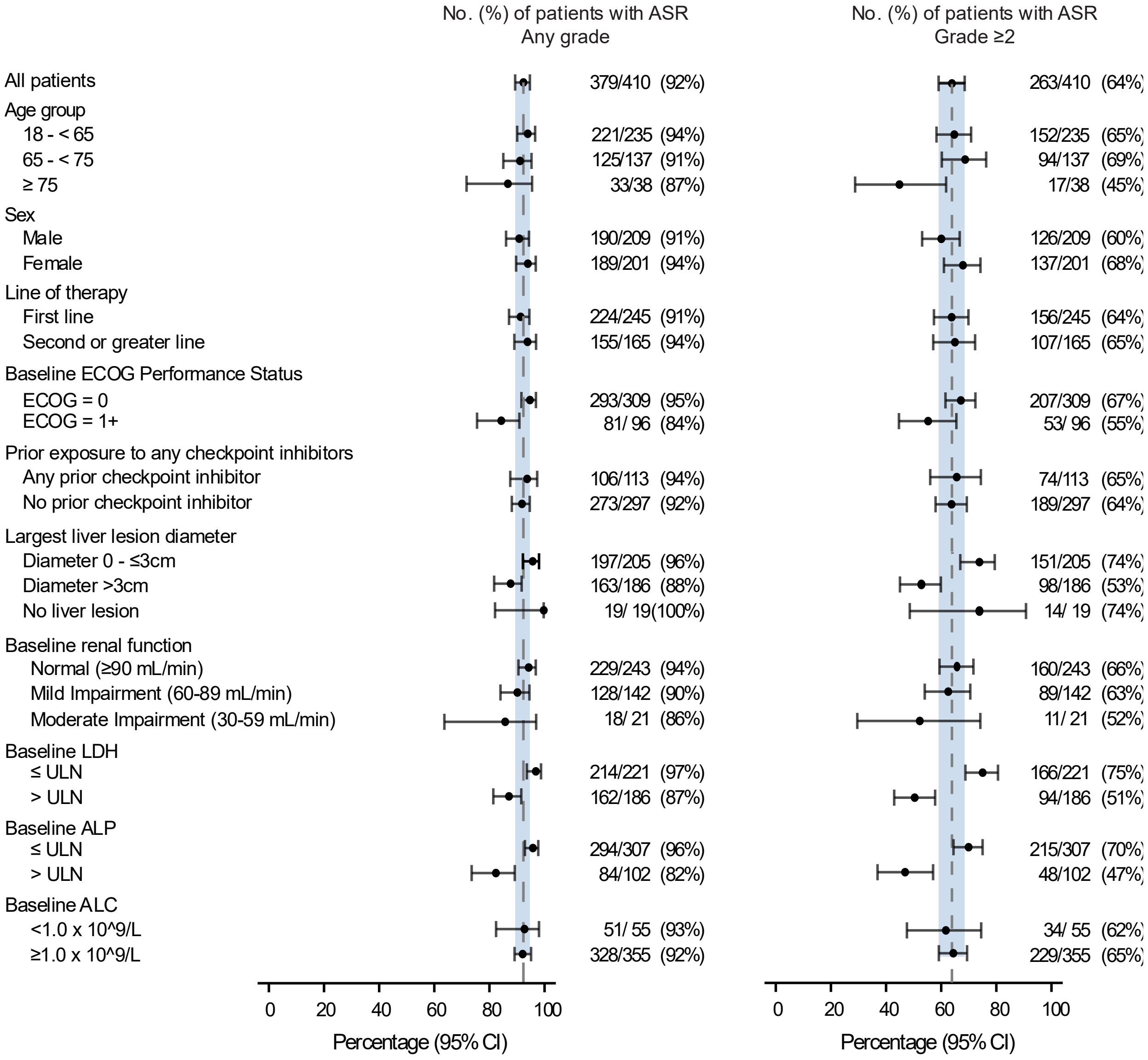


**(B)** Overall incidence of acute skin reactions (ASR; any grade and grade ≥2) by disease characteristic subgroup (N=410). Abbreviations: AESI, adverse event of special interest; CI, confidence interval; ULN, upper limit of normal.

C

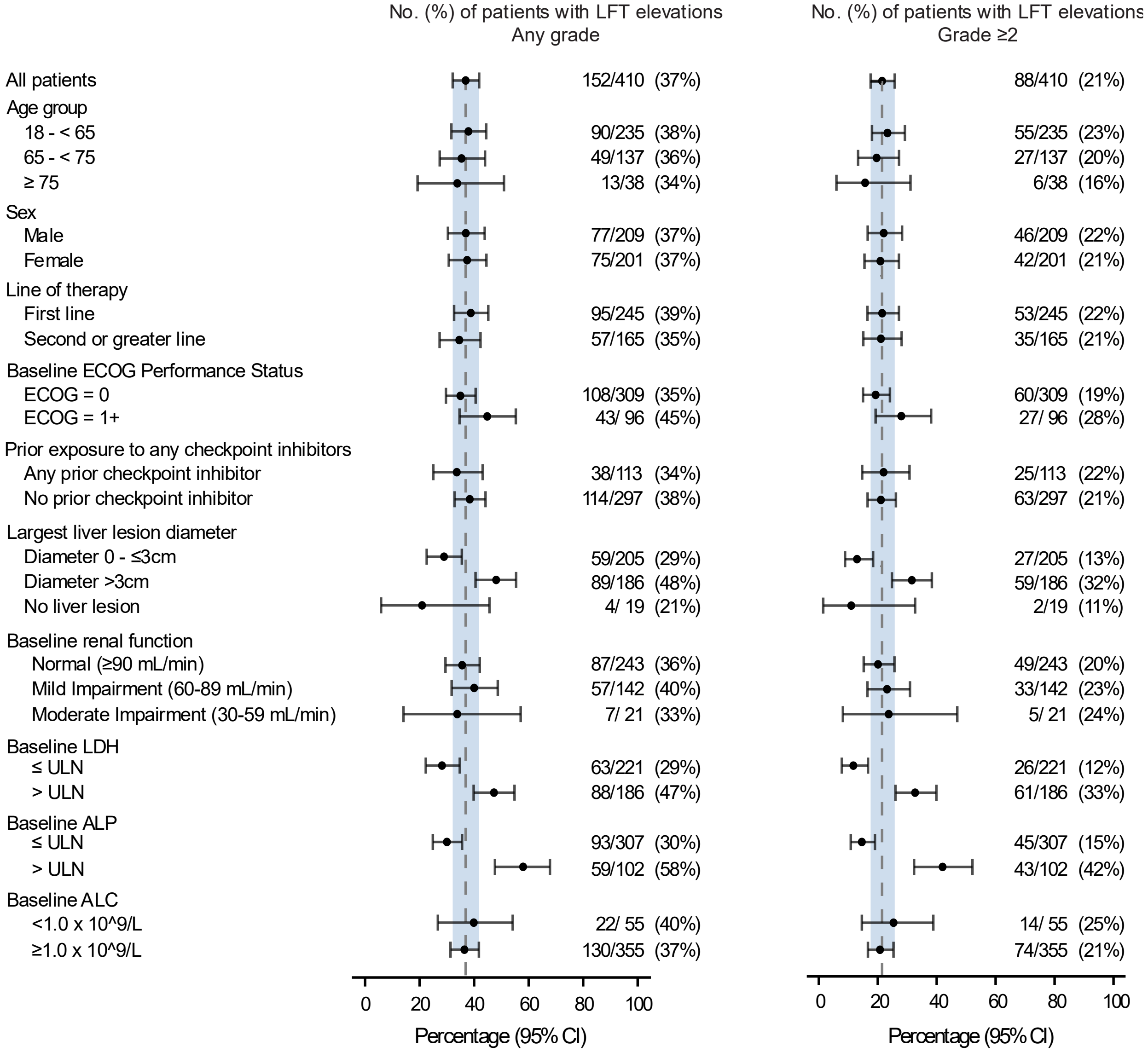


**(C)** Overall incidence of LFT elevations (any grade and grade ≥2) by disease characteristic subgroup (N=410). Abbreviations: AESI, adverse event of special interest; CI, confidence interval; ULN, upper limit of normal.

**Supplementary Figure 2.** Cytokine induction in patients experiencing CRS or rash

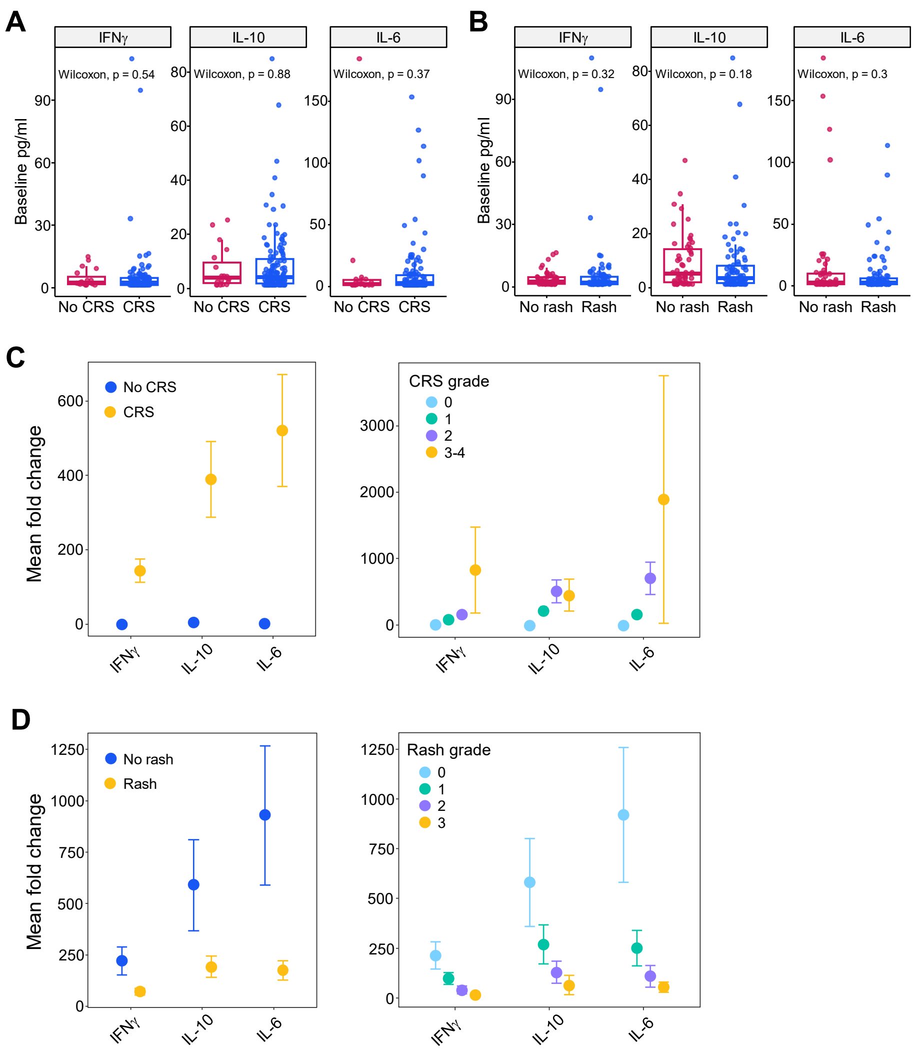


**Supplementary Figure 2. Baseline and on-treatment changes in peripheral cytokines following the first tebentafusp dose.** Baseline and on-treatment changes in peripheral cytokines following the first tebentafusp dose. Concentration of IFNγ, IL-10, and IL-6 were determined at baseline and 8 hrs post first tebentafusp dose in serum samples from 142 2L+ patients in the IMCgp100-102 study. (A & B) Baseline cytokine levels grouped based on presence / absence of (A) CRS or (B) rash. (C) Cytokine release syndrome (CRS) was grouped as maximum CRS grade versus no CRS (left panel) and by CRS grade (right panel), per ASTCT 2019 Criteria. (D) Patients were grouped into rash versus no-rash within 7 days post 1st dose (left panel) and by rash grade (right panel). Data are plotted as mean +/- standard error of the mean.

**Supplementary Figure 3.** Incidence of AESIs over time

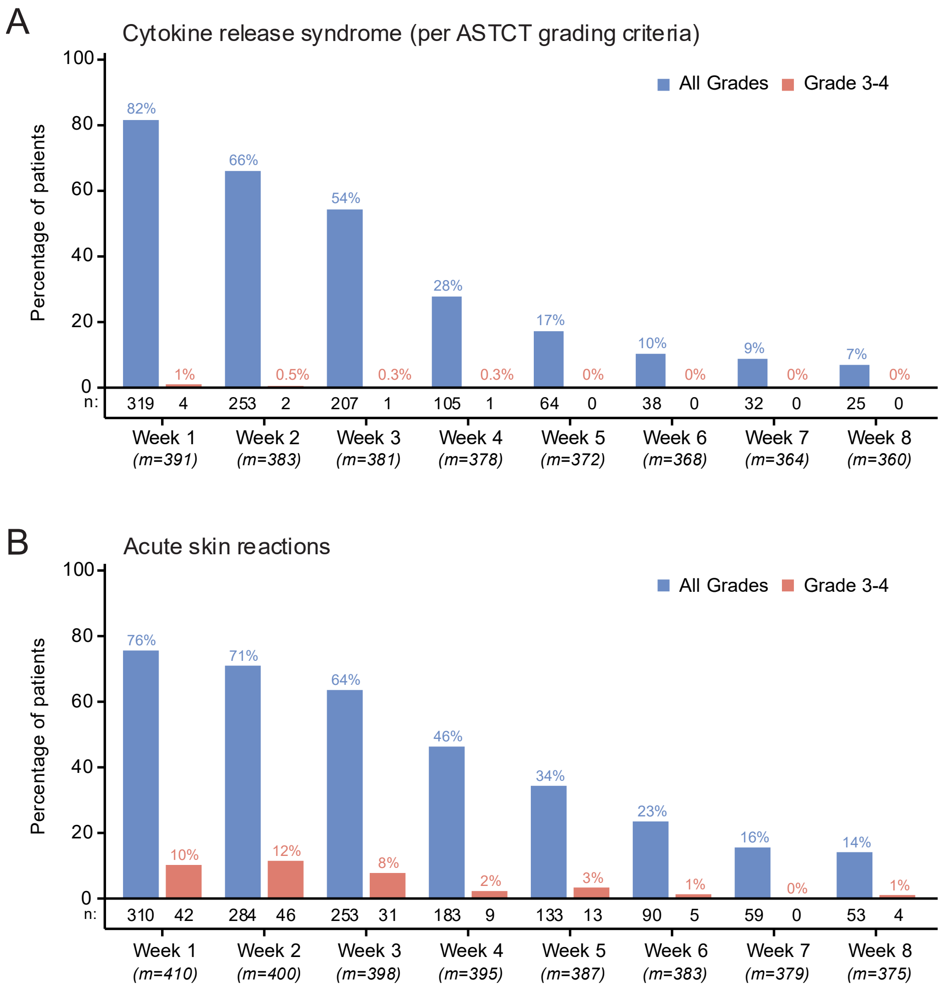


**Supplementary Figure 3. Incidence of AESIs over time.** Incidence of (A) cytokine release syndrome per ASTCT consensus grading criteria and (B) acute skin reactions over the first 8 weeks of treatment with tebentafusp. n denotes number of patients with the AESI and *m* is the number of patients treated.

**Supplementary Figure 4.** Mean change in body vital signs associated with CRS

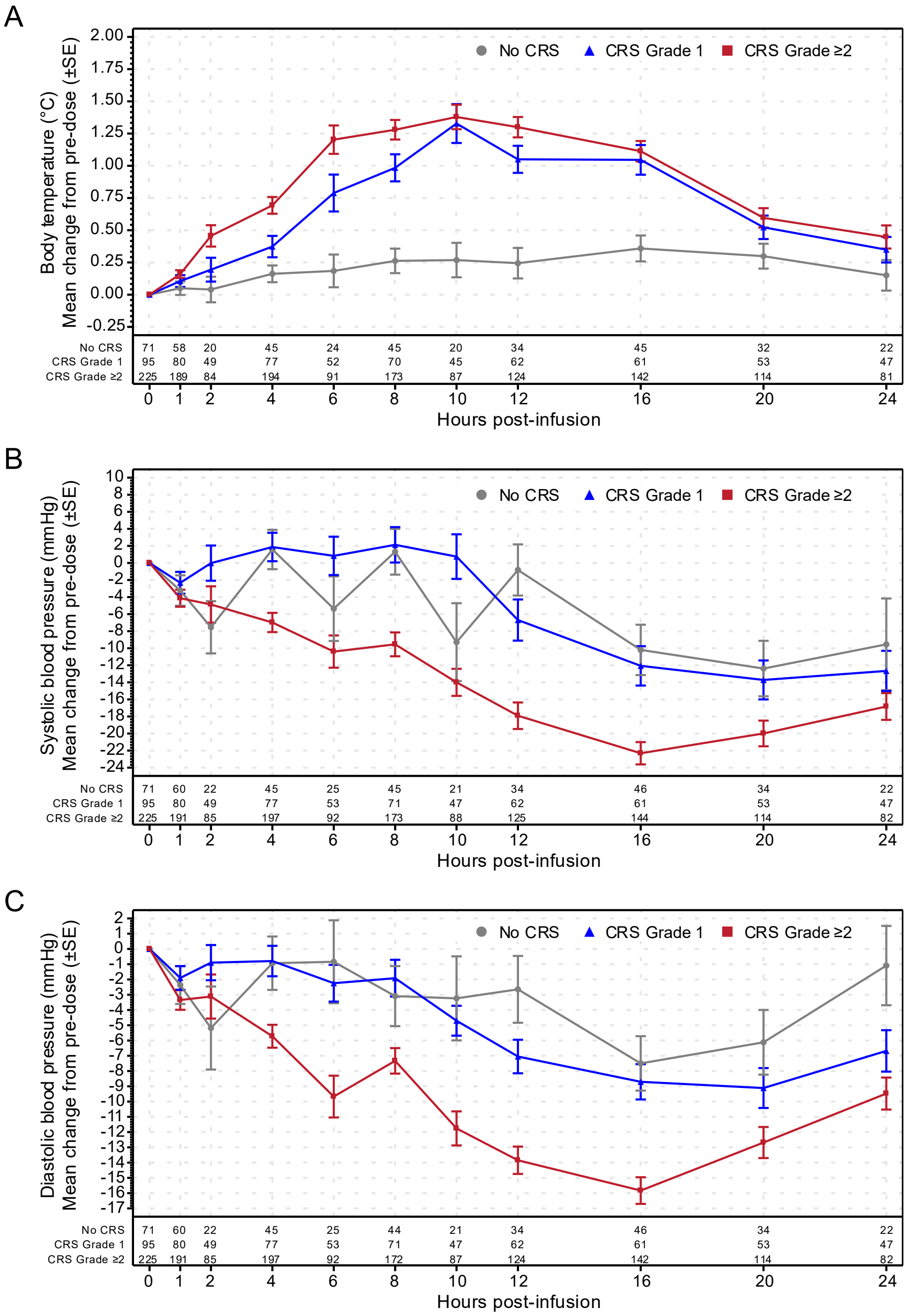


**Supplementary Figure 4.** Mean change from pre-dose **(A)** body temperature (degrees Celsius), **(B)** systolic blood pressure (mmHg), and **(C)** diastolic blood pressure (mmHg) over time following first tebentafusp dose by CRS grade. Includes patients from studies 102 and 202 only. Abbreviations: CRS, cytokine release syndrome.

**Supplementary Figure 5.** Acute skin reaction TRAEs and MRAEs

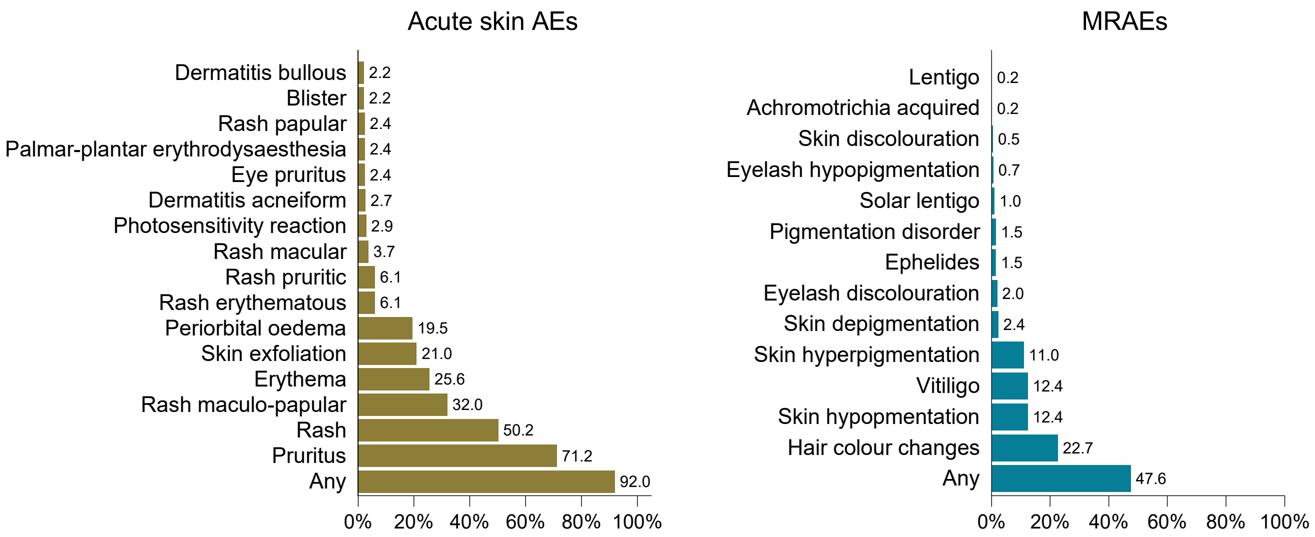


**Supplementary Figure 5.** Frequency of patients with skin TRAEs (≥ 2%) and MRAEs (> 0%) by preferred term.
